# The novel compound heterozygous variants identified in a Chinese family with glucose phosphate isomerase deficiency and pathogenicity analysis

**DOI:** 10.1186/s12920-023-01603-x

**Published:** 2023-07-10

**Authors:** Yang Wang, Tao Liu, Jiaqi Liu, Yan Xiang, Lan Huang, Jiacheng Li, Xizhou An, Shengyan Cui, Zishuai Feng, Jie Yu

**Affiliations:** 1 Department of hematology and oncology, National Clinical Research Center for Child Health and Disorders, Ministry of Education Key Laboratory of Child Development and Disorders, Chongqing Key Laboratory of Pediatrics, Children’s Hospital of Chongqing Medical University, Chongqing, China; 2 Shanghai Cinopath Medical Testing Co Ltd, Shanghai, China; 3 Capital Medical University, Beijing, China; 4 Department of Neonate, Hebei Maternity and Gynecology Hospital, Shijiazhuang, Hebei China

**Keywords:** Glucose phosphate isomerase deficiency, Splicing mutation, Missense mutation, Minigene, Haemolytic anaemia

## Abstract

**Background and Aims:**

Glucose phosphate isomerase (GPI) deficiency is an extremely rare autosomal recessive disorder caused by mutations in the *GPI* gene. In this research, the proband displaying typical manifestations of haemolytic anaemia and his family members were recruited to analyse the pathogenicity of the detected variants.

**Methods:**

Peripheral blood samples were collected from the family members and genomic DNA was extracted and targeted for capture and sequencing. The effect of the candidate pathogenic variants on splicing was further investigated using the minigene splicing system. The computer simulation was also used for further analysis of the detected data.

**Results:**

The proband carried the compound heterozygous variants c.633 + 3 A > G and c.295G > T in the *GPI* gene, which have never been reported before. In the genealogy, co-segregation of the mutant genotype with the phenotype was established. The minigene study showed that intronic mutations resulted in abnormal pre-mRNA splicing. Specifically, the two aberrant transcripts: r.546_633del and r.633 + 1_633 + 2insGT were transcribed by the minigene plasmid expressing the c.633 + 3 A > G variant. The missense mutation c.295G > T in exon 3 resulted in altering glycine at codon 87 to cysteine which was predicted to be pathogenic in an *in silico* analysis. Deeper analyses revealed that the Gly87Cys missense mutation led to steric hindrance. Compared to the wild-type, the mutation G87C led to a significant increase in intermolecular forces.

**Conclusion:**

Overall, the novel compound heterozygous variants in the *GPI* gene contributed to the etiology of the disease. Genetic testing can assist in the diagnosis. The novel gene variants identified in the present study has further expanded the mutational spectrum of GPI deficiency, which can better guide family counselling.

**Supplementary Information:**

The online version contains supplementary material available at 10.1186/s12920-023-01603-x.

## Introduction

Glucose phosphate isomerase (GPI) deficiency is an extremely rare autosomal recessive disorder, and the earliest description is believed to have been in 1968 by Professor Baughuan [1]. To date, no more than 100 cases have been reported worldwide [2]. The exact morbidity rate of GPI deficiency is unknown but is estimated to be less than 1 in 500,000 [3]. GPI is a homo-dimeric enzyme that catalyses the inter-conversion of glucose-6-phosphate and fructose-6-phosphate in the second reaction step of the glycolytic pathway [4]. In addition, GPI has many other features, including as a neuroleukin (a neurotrophic growth factor), myofibril-bound serine protease inhibitor, autocrine motility factor, maturation factor [2, 5]. Consequently, children with GPI deficiency have a broad spectrum of clinical presentations, of which the typical clinical manifestations are mainly anaemia, cholelithiasis, jaundice, dyspnoea, splenomegaly, oedema, or even death in severely affected patients [6]. Defects in GPI synthesis in red blood cells (RBCs) can result in a haemolytic tendency. Over the last decade, with advances in sequencing technology, an increasing number of studies on the genetics and biology of GPI deficiency have been conducted. Ongoing advances in genetics and functional analysis of variants are leading to an increasingly better understanding of the potential pathogenic mechanisms of GPI deficiency.?G and c.295G?>?T in the GPI gene, which have never been reported before. In the genealogy, co-segregation of the mutant genotype with the phenotype was established. The minigene study showed that intronic mutations resulted in abnormal pre-mRNA splicing. Specifically, the two aberrant transcripts: r.546_633del and r.633?+?1_633?+?2insGT were transcribed by the minigene plasmid expressing the c.633?+?3 A?>?G variant. The missense mutation c.295G?>?T in exon 3 resulted in altering glycine at codon 87 to cysteine which was predicted to be pathogenic in an in silico analysis. Deeper analyses revealed that the Gly87Cys missense mutation led to steric hindrance. Compared to the wild-type, the mutation G87C led to a significant increase in intermolecular forces. Conclusion: Overall, the novel compound heterozygous variants in the GPI gene contributed to the etiology of the disease. Genetic testing?can?assist?in?the?diagnosis. The novel gene variants identified in the present study has further expanded the mutational spectrum of GPI deficiency, which can better guide family counselling."?>

Herein, a Chinese Han family with a pedigree of GPI deficiency was recruited. The compound heterozygous variants of the *GPI* gene, c.633 + 3 A > G and c.295G > T, were identified as disease-associated variants using targeted region capture and high-throughput sequencing. A variety of combined bioinformatic tools were also subsequently utilised to assess the effects of these mutations on protein function. Furthermore, in vitro minigene assays were performed to demonstrate the effects of the splicing mutation.

## Materials and methods

### Patient

**Fig. 1:**
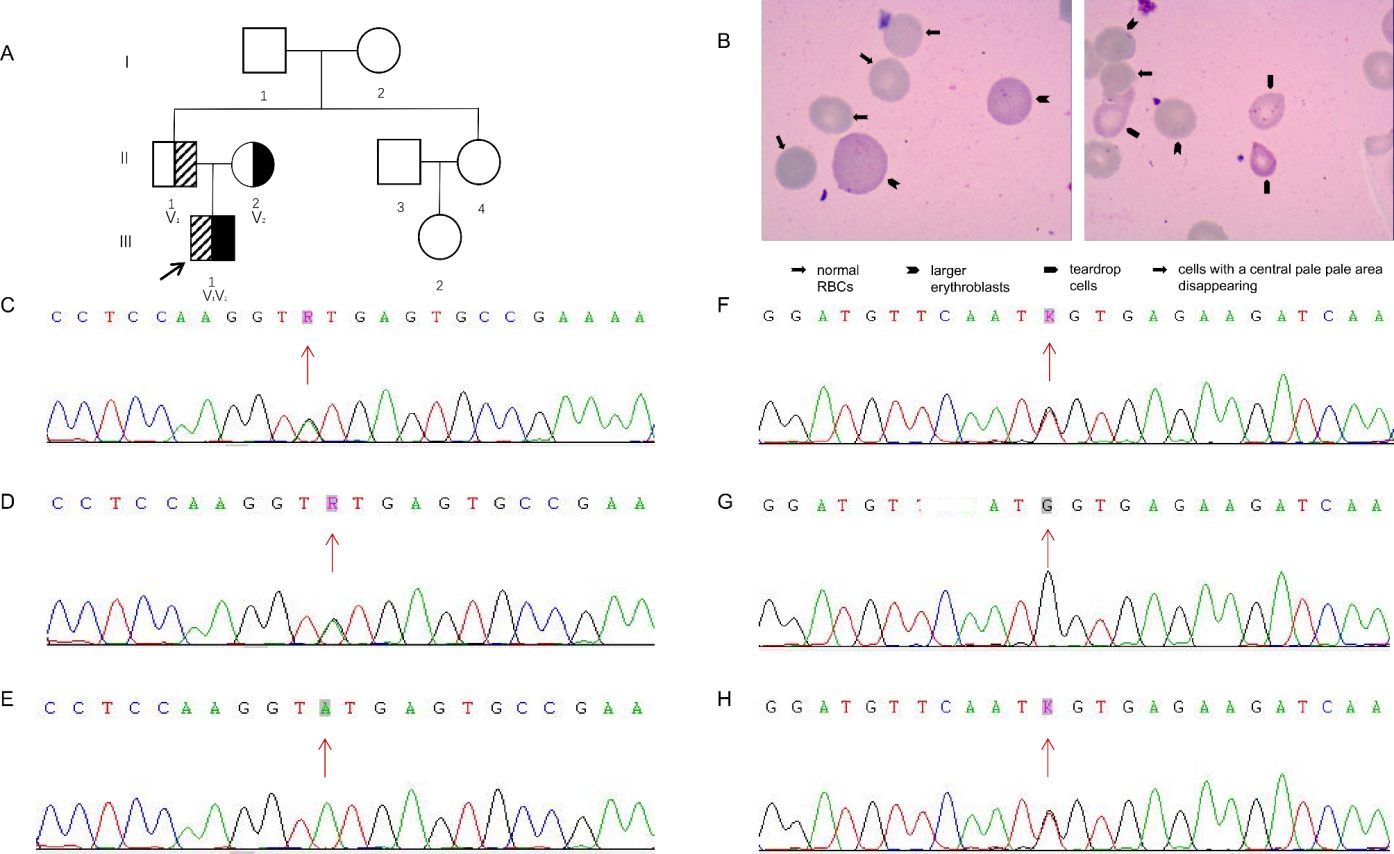
GPI deficiency in the pedigree and the verification results of Sanger sequencing. **(A)**: Third-generation kindred in the pedigree and associated *GPI* genotype based on genetic testing data and clinical presentation. The shaded square and variant 1 (V1): c.633 + 3 A > G variant; solid filled black and variant 2 (V2): c.295G > T variant. The arrow represents the proband. The compound heterozygous variants, V1 and V2, in the GPI gene of the proband inherited from the father (II-1) and the mother (II-2), respectively. **(B)**: Peripheral blood smears of the proband. **(C)**: The c.633 + 3 A > G variant in the *GPI* gene of the proband. **(D)**: The same variant in the father. **(E)**: No mutations were found in the mother. **(F)** The exon 3: c.295G > T variant in the *GPI* gene of the proband. **(G)**: No mutations were found in the father. **(H)**: The same variant in the mother

A 4-year-old male toddler was admitted to our hospital for anaemia that had persisted for approximately 3 years with complaints of progressive jaundice for 5 days. The proband had a normal birth and his parents were non-consanguineous and had no history of congenital anomalies or recurrent miscarriages. Physical examination revealed scleral jaundice, pallor, and consciousness. A clinical professional assessment revealed that the child was developmentally similar to his age peers and matched at the grammatical comprehension level; he could practice self-care, such as toileting, eating, dressing, and bathing without assistance. This patient had a palpable liver (5 cm below the costal margin and 6 cm below the xiphoid) and the spleen was noted below the costal margin. His laboratory findings were as follows: haemolytic anaemia (haemoglobin [Hb]: 83 g/L; mean corpuscular volume: 95.7 fL, mean corpuscular haemoglobin: 30.1 pg, mean corpuscular haemoglobin concentration: 309 g/L, haematocrit: 26.9%, red cell distribution width: 19.2%, reticulocyte count 229.5 × 10^9^/L, and reticulocyte percentage 6.2% and unconjugated hyperbilirubinaemia (total bilirubin: 86.6 µmol/L; indirect: 85 µmol/L). He also presented with high lactate dehydrogenase (654 U/L) and low serum haptoglobin levels (0.91 g/L). The serum ferritin level of the proband was 895 ng/mL, which was well above the reference values (the normal range is 20–200 ng/mL). The heterogeneity of the mature erythrocyte size, polychromatophilic erythroblasts, and basophilic erythroblasts was observed using light microscopy (Olympus Optical, Tokyo, Japan). The blood cell morphology is shown in Fig. 1B. GPI, pyruvate kinase, and glucose-6-phosphate dehydrogenase activities of RBCs were measured and all results were normal. Evaluation of ultrasonography of the abdominal revealed that the right hepatic lobe oblique diameter was 8.8 cm and the spleen was 1.1 cm below the left costal margin, the long diameter of the spleen was about 9.8 cm, and the thickness was about 2.6 cm. In 2017, after a cardiopulmonary bypass, atrioventricular septal defect repair had been operated on, and the patient received an intraoperative transfusion of RBCs. Collectively, the clinical presentations and laboratory findings supported the diagnosis of haemolytic anaemia, and inherited metabolic diseases were considered.

### Family survey and sample collection

Detailed demographic information was obtained from the available members of the family including age, sex, and disease onset, and then the pedigree chart was mapped. Peripheral blood was collected for gene sequencing.

### Whole exome sequencing of the *GPI* variants

Whole genomic DNA was extracted from peripheral blood, and the target regions of disease-associated genes were captured and sequenced at an average depth of 500–1000× according to the manufacturer’s instructions. IDT xGen Exome Research Panel v1.0 capture kit, KAPAHiFi Ready Mix enzyme, splice and index primers, and other reagents (Vazyme Biotech, China) were used for DNA library construction. Nearly 700 genes for hereditary diseases of the blood and immune system were screened using NovaSeq 6000 equipment (Illumina, USA) to detect variants. Candidate pathogenic mutations detected were verified by Sanger sequencing, and the results were sequenced in both directions using an automated sequencer 373XL DNA Analyzer (Applied Biosystems, USA) and analysed.

### Bioinformatic analysis of the c.633 + 3 A > G and c.295G > T variants

The mutations were compared to those in the 1000 Genomes Project, and then the following tools were used for gene annotations: sorting tolerant from intolerant (SIFT) (https://sift.bii.a-star.edu.sg/), polymorphism phenotyping v2 (PolyPhen-2) (http://genetics.bwh.harvard.edu/pph2/), Mutation Taster (HTTP://www.mutationtaster.org/), and rare exome variant ensemble learner (REVEL) (https://sites.google.com/site/revelgenomics/). The effect of the mutation on pre-mRNA splicing was evaluated using the Human Splicing Finder (HSF, https://www.genomnis.com/access-hsf), Splice AI (https://spliceailookup.broadinstitute.org/) and RDDC RNA Splicer (https://rddc.tsinghua-gd.org/). In parallel, the protein encoded by c.295G > T modelling was achieved by PyMOL Molecular Graphics System (Version 2.5.4) [7]. PDB ID 1IRI was chosen as the template for visualising the three-dimensional structure. Additionally, we used DynaMut webserver to visualise and assess the stability and interactions of the mutant protein [8]. The following information was fed into DynaMut: wild-type (WT) structure (PDB accession code: 1IRI) and variant detail (G87C, chain A). The detailed methodology has been previously described [9].

### In vitro splicing of minigene assay of c.633 + 3 A > G variant

To investigate the effect of the c.633 + 3 A > G variant on mRNA splicing, an in vitro experimental validation was conducted. The WT plasmids and mutant plasmids were constructed using human genomic DNA as templates. After Sanger sequencing verification, the amplified fragments were reconstituted into pMini-CopGFP (with BamHI and XhoI sites in the vector) using ClonExpress II One Step Cloning Kit (Vazyme, Nanjing, China). Next, the products were used directly for the transformation of competent TOP10 cells, and positive colonies were selected and amplified. Next, 293T cells density was adjusted to 5 × 10^5^ using the Automated Cell Counter IM1200 (Countstar, Shanghai, China) and transfected by the plasmids following instructions for Lipofectamine 2000 Transfection Reagent (Invitrogen, #11668-019) following the instructions. In the final step, the RNA sample was extracted from cells cultured for 48 h using TRIZOL reagent and subjected to a reverse transcription-polymerase chain reaction (RT-PCR). All the primer-related information and PCR conditions are described in Supplementary Material 1.

## Results

### Pedigree and genetic analysis

In the pedigree, the proband exhibited anaemia and the rest of the family had normal haemoglobin levels. High-throughput sequencing results revealed compound heterozygous variants NM_000175.5: c.633 + 3 A > G and NM_000175.5: exon 3: c.295G > T in the *GPI* gene, which was not found in the GNOMAD database (http://gnomad-sg.org/). Sanger sequencing confirmed the co-segregation of mutations with the phenotypes in this family. The results are shown in Fig. 1.

### Bioinformatic analysis and experimental validation of the minigene assays of c.633 + 3 A > G

**Fig. 2:**
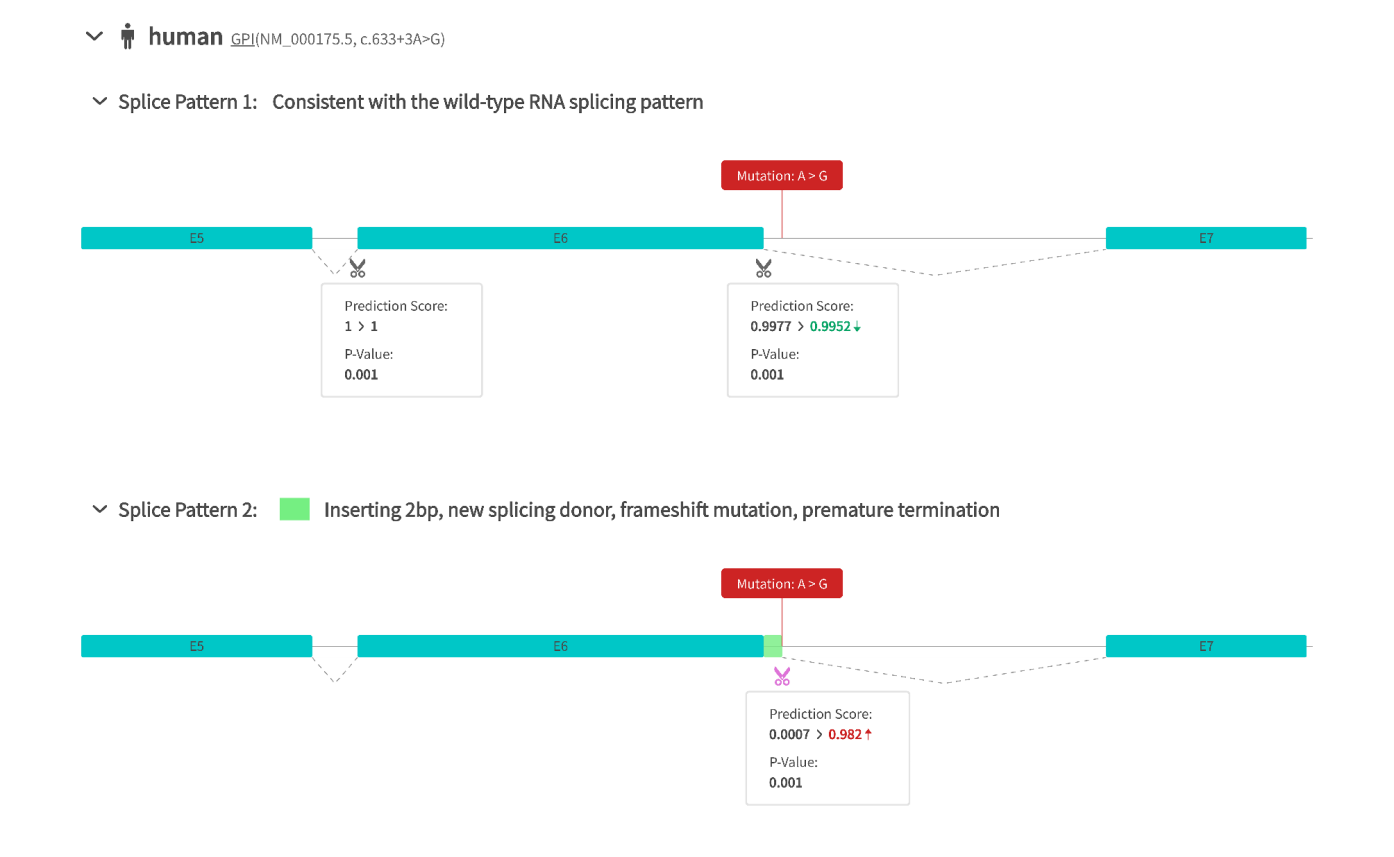
RDDC RNA Splicer prediction results. The c.633 + 3 A > G variant can produce two splice forms, including the wild-type RNA splicing pattern and a 2 bp insertion, providing a new splicing donor

To the best of our knowledge, the c.633 + 3 A > G variant located in the + 3 position of exon 7, which resulted from an A > G base change. The function of the c.633 + 3 A > G variant of the *GPI* gene was predicted as unknown by three protein function prediction software (SIFT, PolyPhen2, and REVEL). However, the splicing prediction software suggested that the mutation affected the “mutation-II region” of splicing and that it might lead to abnormal splicing of exons [10]. The HSF revealed that the original donor site was disrupted and a new donor site was created after the mutation, suggesting that the mutation affects splicing. Splice AI predicted that the c.633 + 3 A > G variant could lead to donor gain with a score of 0.96 (at −1 bp distance) and donor loss with a score of 0.01 (at −3 bp distance). In our study, 50 bp was the maximum distance for Splice AI predictions (see Fig. 2 for the details). Significantly, the c.633 + 3 site of the *GPI* gene is highly conserved (Fig. 4A).

**Fig. 3:**
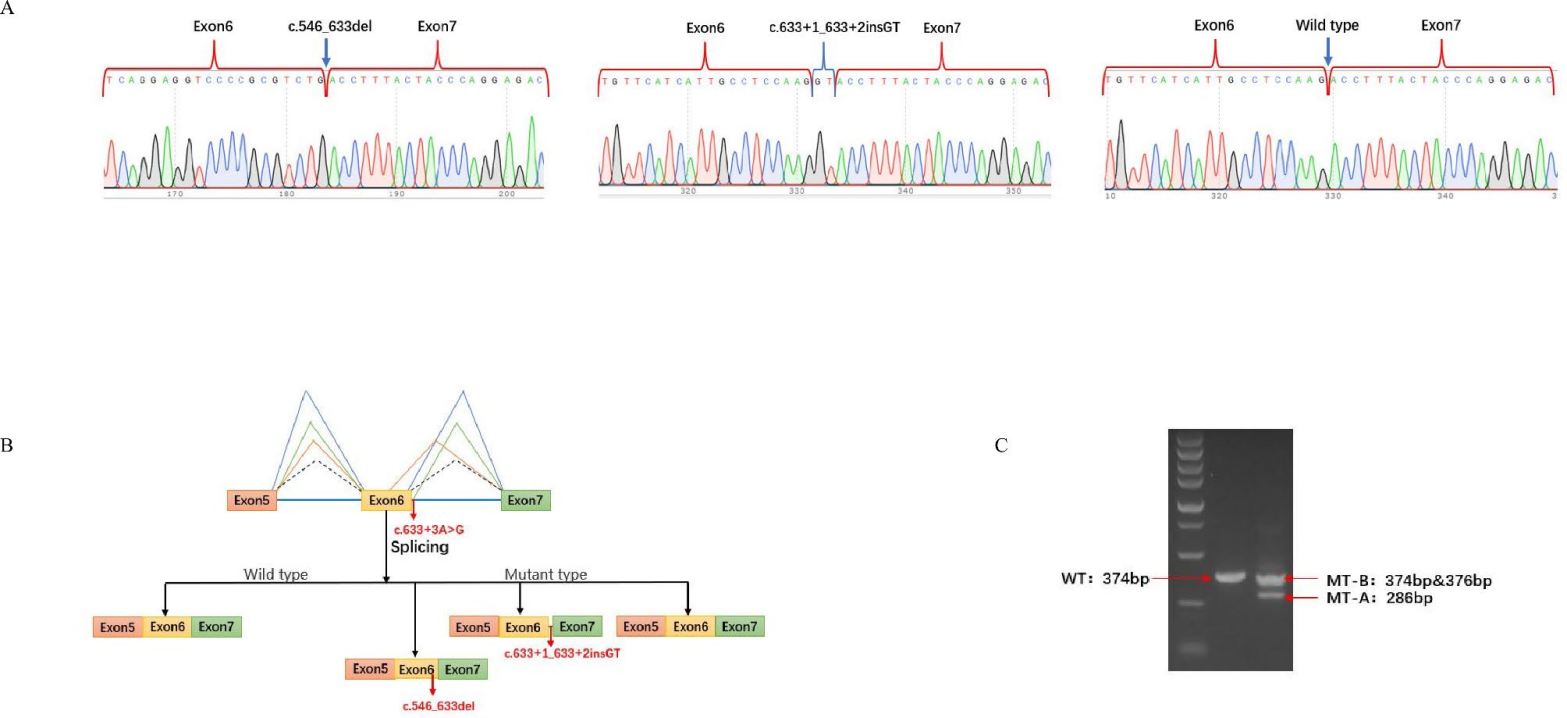
Minigene assay for the *GPI* c.633 + 3 A > G variant and schematic diagram of the splicing pattern. **(A)** Sanger sequencing chromatograms of the reverse transcription-polymerase chain reaction (RT-PCR) products of the c.633 + 3 A > G variant. **(B)** Schematic diagram of the wild-type (WT) and mutated minigene fragments. The ttranscribed mRNA sequence of the WT plasmid was consistent, including complete exons 5, exon 6 and exon 7. The three transcripts: r.546_633del, r.633 + 1_633 + 2insGT and WT sequences were transcribed by the minigene plasmid. **(C)** Agarose gel electrophoresis of the RT-PCR products of the WT and mutated minigenes of the c.633 + 3 A > G variant. The original picture is in Supplementary Material 3

A similar, but not identical, result was obtained when from the experiments. The following are some details. The DNA sequence determined was 374 bp in the standard. The mRNA sequences encoded by WT plasmids were as expected. Meanwhile, the amplification products sizes were 374 bp, 376 bp, and 286 bp in 293 T cells transfected with the c.633 + 3 A > G mutant plasmid. The transcription products of the minigene assay were visualised by agarose gel electrophoresis. The results of these analyses are shown in Fig. 3. Next, we used an online tool (https://en.vectorbuilder.com/tool/dna-translation.html) to translate the different transcripts into amino acid sequences. The amino acid sequences, presented in Supplementary Material 2, were very different from the WT sequences.

### Bioinformatic analysis of c.295G > T

**Fig. 4:**
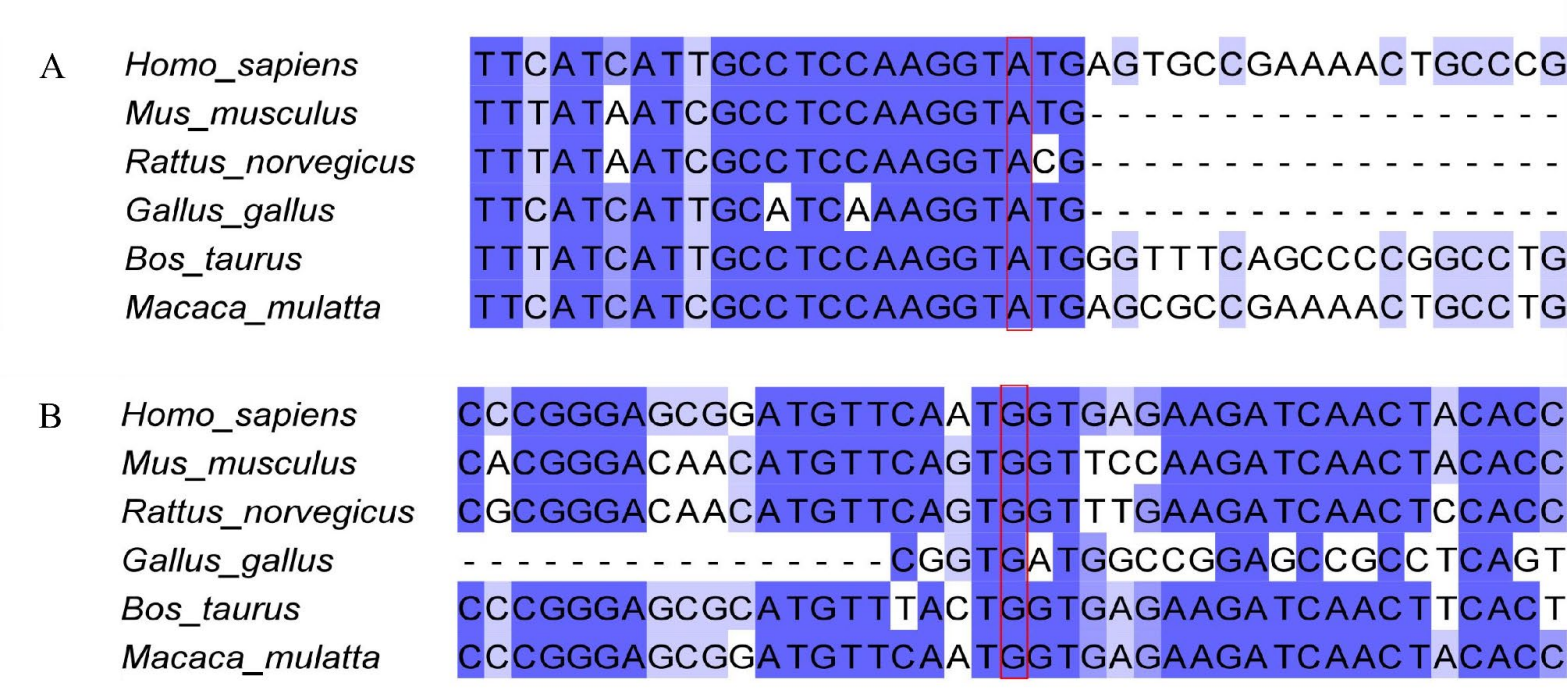
Multiple sequence alignments of the site variant of the *GPI* gene from Homo sapiens, Mus musculus, Rattus norvegicus, Gallus, Bos Taurus, and Macaca mulatta revealed a high degree of evolutionary conservation. Conserved residues are shown in red boxes

**Fig. 5:**
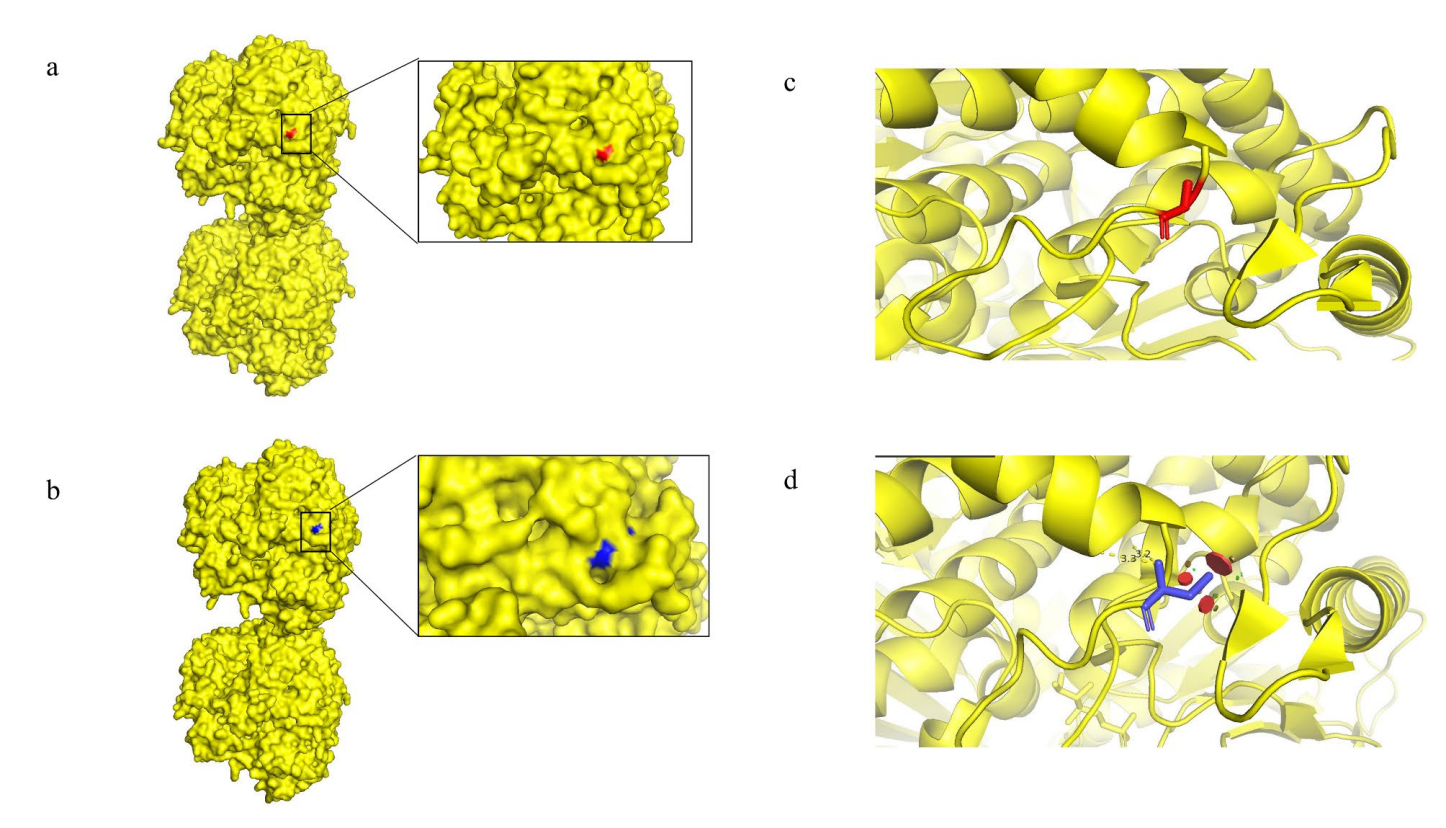
Modelling of the wild-type and mutant-type protein of GPI. **(a, b)** Three-dimension (3D) structure of the GPI protein. Residue 87 is marked in red and the G87C is marked in blue. **(c, d)** A 3D structure showing before and after mutation of residue 87 (G87C) in a cartoon format. The wild-type and mutant-type proteins are coloured red and blue, respectively; the mutant G87C protein reveals the steric hindrance

**Fig. 6:**
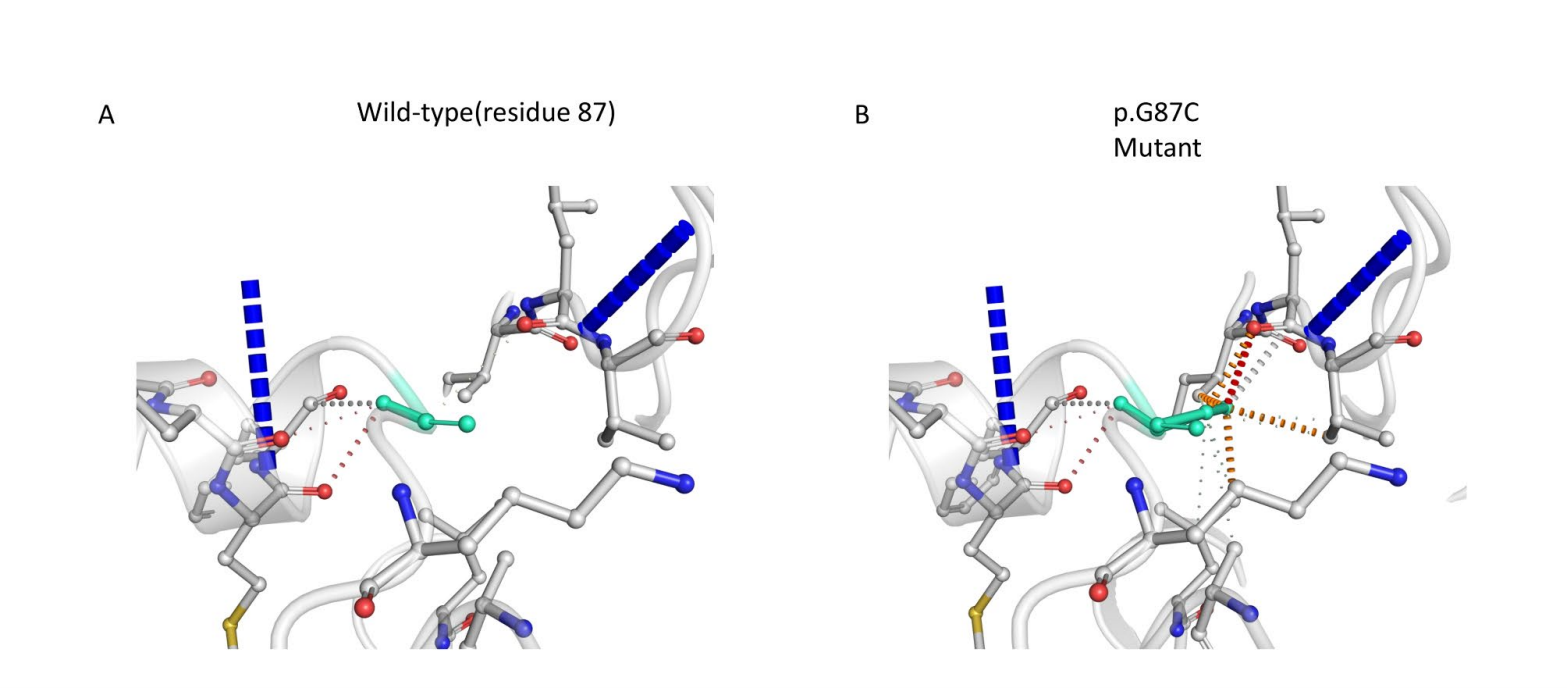
Predictive interactions of the wild-type and mutant sites of GPI protein. Residues in the wild-type and mutant sites are coloured light green and shown as sticks. The respective chemical interactions are labelled as dotted lines and coloured as follows: hydrogen bonds—red; weak hydrogen bonds—orange; hydrophobic contacts—green; amide-amide contacts—blue and ionic interactions—gold. Amino acid residues are also coloured according to type, namely nitrogen (blue), oxygen (red), and sulphur (yellow). Compared to the wild-type site, some interactions (hydrophobic, amide-amide, and ionic bonds) were observed to increase in the mutant site

The c.295 site of *GPI* is highly conserved among species (Fig. 4B). The c.295G > T variant in exon 3 led to amino acid replacement at position 87(G87C). SIFT score of 0, PolyPhen2 score of 0.988, and REVEL score of 0.894 were derived from the protein function prediction software. In our study, the PyMOL and DynaMut were used to visualise mutations in proteins. Comparison with the WT protein shows that the c.295G > T variant leads to steric hindrance (Fig. 5). From further analysis, we found that the mutant site showed an increase in specific molecular linkages (Fig. 6 legend for more details).

## Discussion

GPI deficiency is an uncommon genetic disorder that follows an autosomal recessive pattern of inheritance. The patient with GPI deficiency typically carries either a homozygous or compound heterozygous genotype [5]. Conventional laboratory tests are insufficient to provide specific indications beyond heamolytic anaemia for the diagnosis of GPI deficiency. The diagnosis is primarily made by excluding other common causes of congenital heamolytic anaemia, demonstrating decreased GPI enzyme activity in RBCs, and confirming the diagnosis through genetic testing. In recent years, with the advancement of genetic testing, an increasing number of cases of GPI deficiency have been identified. In our study, we identified novel compound heterozygous variants of the *GPI* gene (c.633 + 3 A > G and c.295G > T) which were responsible for GPI deficiency. To explore the functional consequences of these mutations, we employed computational and experimental approaches.

Using “glucose phosphate isomerase deficiency” as the keyword for searching the online databases, PubMed, CNKI, and Wan Fang Med Online, a total of 367 articles were retrieved as of August 2022. Overall, 66 publications were included after removing duplicates; these comprised 89 cases of GPI deficiency in 44 male and 45 female individuals. The age of onset of chronic macrocytic anaemia is typically at birth or early infancy. To date, the most extensive single-centre study has been done in India, which enrolled 17 patients with GPI deficiency [5]. Furthermore, Professor Fermo’s study in Italy had the longest follow-up period with a median follow-up of 18 years (ranging from 2 to 40 years) for patients with GPI deficiency to date [2]. Clinical manifestations of GPI deficiency including reticulocytosis, jaundice, and splenomegaly associated with mild hepatomegaly reflected individual differences between patients [11]. Chronic non-spherocytic heamolytic anaemia of varying severity is the only clinical manifestation of most patients with GPI deficiency. Additionally, a minority of patients with GPI deficiency suffered from neuromuscular dysfunction, foetal oedemas, and neonatal deaths [2]. Patients with GPI deficiency had recurrent infections, which induced a haemolytic crisis [12]. The child presented with jaundice, anaemia, and hepatosplenomegaly from birth. Moreover, laboratory examinations indicated hyperbilirubinaemia, elevated lactate dehydrogenase levels, and macrocytic and hypochromic anaemia, which is consistent with previous findings. Notably, neuromuscular impairment was absent in this case, indicating phenotypic diversity and complexity in GPI deficiency.

Located on human chromosome 19q13.1, the *GPI* gene transcribes to a 1.9 kb cDNA containing 18 exons that translates to a 558 amino acid protein, which is a member of the phosphoglucoisomerase family [11]. The *GPI* gene encodes glucose-6-phosphate isomerase, which catalyses the cytoplasmic conversion of glucose-6-phosphate to fructose-6-phosphate, is involved in the second step of the sub-pathway that synthesises D-glyceraldehyde 3-phosphate and glycerone phosphate from D-glucose. Thus, glucose-6-phosphate isomerase enzyme deficiency correlates with the accumulation of glucose-6-phosphate and lack of ATP, which ultimately leads to RBCs lysis [13]. Furthermore, the *GPI* gene encodes products with cytokines activity and neuro-nutritional effects are similar to that of neuroleakin [14]. GPI activity assay is the diagnostic method for this disease, although GPI activity is susceptible to factors such as blood transfusion, leading to false negative results. Genetic testing is currently the most reliable way to confirm the diagnosis [6]. In the current study, the proband’s GPI enzyme activity was a false normal because of blood transfusion therapy, which cluttered the diagnosis of GPI deficiency on admission. After the genetic results suggested the possibility of GPI deficiency, the GPI enzyme activity was re-evaluated after suspending the transfusion for 3 months, and the results showed a decrease in enzyme activity to 33.1 EU/g Hb, significantly lower than the normal (55–72 EU/g Hb) value. We measured the GPI enzyme activity of the proband’s parents during the same period. GPI enzyme activity was lower than normal in both groups, with the father’s measurement as 49.7 EU/g Hb and the mother’s as 50.3 EU/g Hb. Therefore, in combination with the clinical presentation, the diagnosis was confirmed. In cases of congenital haemolysis of unknown aetiology, it is necessary to consider the possibility of a GPI deficiency. The GPI enzyme activity test should be performed before the start of transfusion therapy, and if necessary, the *GPI* gene test should be performed to assist in the diagnosis.

To date, over 50 GPI deficiency-associated mutations have been reported, the majority of which are missense variants. Specifically, the worldwide spectrum of *GPI* variants shows that c.1040 G > A (p.Arg347His) homozygous mutation in exon 12 of the *GPI* gene is the most common [15]. The c.633 + 3 A > G and c.295G > T compound heterozygous mutations in the *GPI* gene have been reported for the first time from China by our group in this study. Additionally, the c.633 + 3 A > G alternative splicing generated multiple transcripts, which were predicted to be pathogenic by the in vitro splicing assay using the minigene. Moreover, the protein encoded by the *GPI* gene contains 24 α-helices, 8 β-folds, and 33 random coils, of which the percentage of secondary structure is 42.64% for α-helix, 8.49% for β-fold, and 48.87% for random coils. The c.295G > T mutation in exon 3 is a missense variant located in the protein-coding region, and it is speculated that this variant may lead to functional alteration. When specified, the missense variant caused amino acid substitution at position 87. The hydrophobic glycine was mutated to a hydrophilic cysteine which occurred at the secondary structure helical domain. Note that the hydrophobic amino acids are concentrated on one side of the helix and the hydrophilic amino acids on the other. A modification in the polar and non-polar amino acids might result in a change in the protein structure. DynaMut was used to further assess the effect of missense mutations on proteins [9]. According to the prediction results, the G87C mutation is likely to create additional steric hindrance at the binding site, thus preventing substrate binding [16]. As reported by Manco L et al., the structural change induced by the p.Gly87Ala pathogenic variant has a direct impact on the structural arrangement of the region close to the active site of the enzyme [17]. In addition, amino acid substitution increased intermolecular forces. Thus, it is most likely that the c.295G > T mutation works through gain-of-function mechanisms. Further experimental definitive proof of this hypothesis is required. Based on these results, the c.633 + 3 A > G and exon 3: c.295G > T compound heterozygous mutations form the molecular basis of GPI deficiency.

The patient is currently in good condition with regular follow-up, and the haemoglobin can be presently maintained at around 110 g/L without transfusion. Splenectomy may benefit patients with transfusion-dependent GPI deficiency patient [5]. Prophylactic vaccination prevents an infection-induced fatal haemolytic crisis; however, the timing and mode of vaccine administration must be considered with care [2]. Genetic counselling and prenatal diagnosis are necessary when a patient is ready to consider pregnancy again. Further study is warranted to explore the molecular mechanisms in the future.

## Conclusions

In summary, the clinical presentation of GPI deficiency is very similar to that of other haemolytic diseases. At the same time, GPI deficiency is rare in clinical practice. We recommend early genetic testing for a definitive diagnosis, especially in cases of recurrent haemolytic anaemia with unexplained common aetiology. The novel *GPI* gene mutations in this study has enriched the gene mutation database and provides a reference for genetic counselling as well as for more advanced studies.

## Electronic supplementary material

Below is the link to the electronic supplementary material.

Supplementary Material 1

Supplementary Material 2

Supplementary Material 3

## Data Availability

The data analysed in the current study are available in the GenBank database. The Gene ID of *GPI* used in the current study is 2821. The cDNA sequence number is NM_001289789 and the gDNA sequence number is NG_012838.3.

## Abbreviations

GPI: glucose phosphate isomerase
RBC: red blood cell
WT: wild-type
