# Supplementary material for: The novel compound heterozygous variants identified in a Chinese family with glucose phosphate isomerase deficiency and pathogenicity analysis
Source: BMC Med Genomics. 2023 Jul 10;16:162. doi: 10.1186/s12920-023-01603-x (PMC10332073; doi:10.1186/s12920-023-01603-x)
Supplement: Supplementary file 1 — Supplementary Material 1 [file 12920_2023_1603_MOESM1_ESM.doc]

**Supplementary material 1.**

Detailed methods and results of the *in vitro* minigene experimental validation.

1. **Diagram of the backbone of pMini-CopGF expression plasmid.**


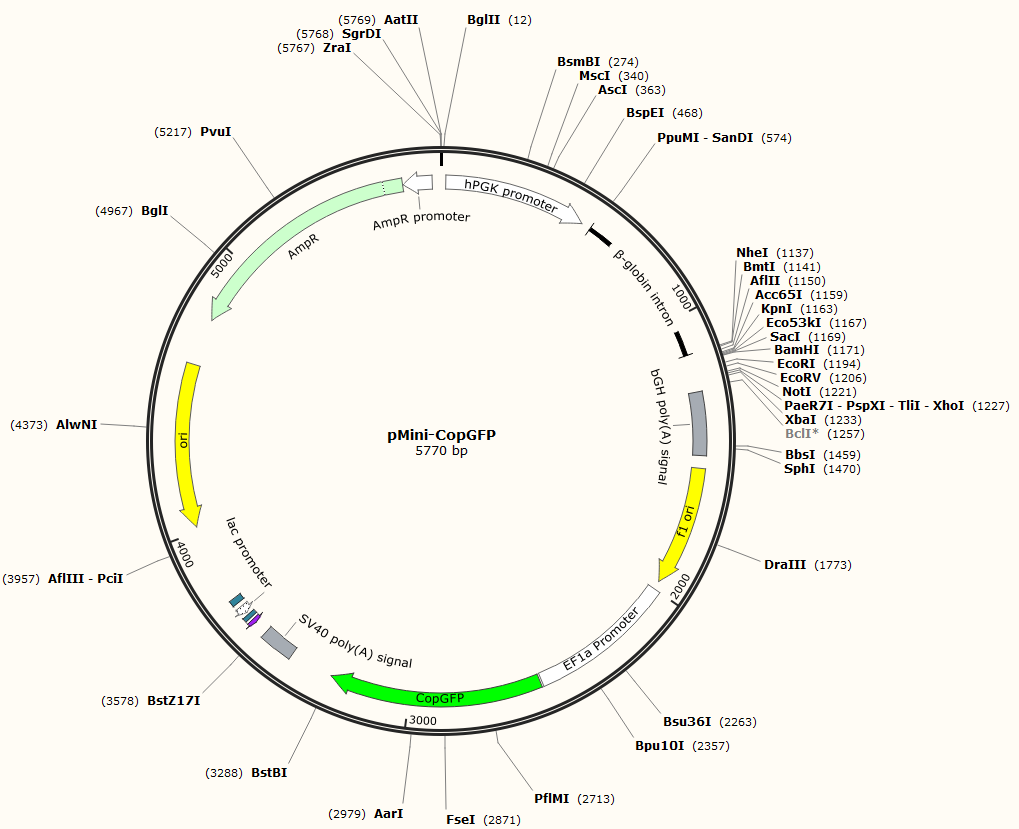


**2. Plasmid Vector Construction.**

Vector: pMini-CopGFP

Restriction enzyme cutting sites: BamHI/XhoI

**3.Inserted sequences**

**3.1. GPI-WT**

cgtgtccggagcggtgactggaaggggtacacaggcaagaccatcacggacgtcatcaacattggcattggcggctccgacctggtgaggagaaaactgccttggggtagggtgggagtctgggcactgttggtcccactcaggtctttactttctccagggatgggacctggctgtctccacttttcgtgggccctgaattcttattctctgatgctatgtctccccgcagggacccctcatggtgactgaagcccttaagccatactcttcaggaggtccccgcgtctggtatgtctccaacattgatggaactcacattgccaaaaccctggcccagctgaaccccgagtcctccctgttcatcattgcctccaaggtatgagtgccgaaaactgcccggcccctggccctgtgtgtgttggggtggggagggacagctgtcttgccatccccctggccattggtcccttttggtgggttccgagtgaaccatggtttgtggatcaggtcagtacagctgccctagactgtttccatgcctagcagcaaataaggttgactgatgaaatcctgaacacatcagtttgaacctttgtgtcctgaccacacccgctcgccttgcggcatctctgctcagcctgggactgctcgccactttcttacaaacctgtcctttatgcctgctccttttcttgttgttgtttttgagacagagtctggctctgtcacccaggctggagtgtagtggcgtgatctcggctcactgcaatctccgtctcccaggtttcaagcgattctcctgcctcagcctcccaagtagctgggactaggcacacaccaccacgcctggctaattttattatttatttatttattattattattttttagtagagatgggcttttgccatgttggccatgctggtcttgaactcctgacctcaggtgatgcacccccccttggcctcccaaagtgctgggattacagtcttgagccgccatgcccagccatgcttgctcctttctgatcccagagccatgccctgataatagaggggttttgtttgtttttatttatgttctatttactcacatgaatgtacataaaatatgaagaattccatctgaattctgagataccttccagcctgggccgaagagagcctcaggttgactgcagcccctcaggggagaagctgcggcctttgacctgcaggcttagggttggggggtgtgtttaccgtcccccctcctctggttttaaagagctggaatctcaggaggttatgtggcgtcactgtcactgacctgcaaatactgctccatgggacagctgggcattgccttggcctctactgctgaaccctggctcaaggcctgcacccacccctaagctcgggcgcccactgctgttctctttggttgcagacctttactacccaggagaccatcacgaatgcagagacggcgaaggagtggtttctccaggcggccaaggat

**3.2. GPI-MT(NM_000175.5：c.633+3A>G)**

cgtgtccggagcggtgactggaaggggtacacaggcaagaccatcacggacgtcatcaacattggcattggcggctccgacctggtgaggagaaaactgccttggggtagggtgggagtctgggcactgttggtcccactcaggtctttactttctccagggatgggacctggctgtctccacttttcgtgggccctgaattcttattctctgatgctatgtctccccgcagggacccctcatggtgactgaagcccttaagccatactcttcaggaggtccccgcgtctggtatgtctccaacattgatggaactcacattgccaaaaccctggcccagctgaaccccgagtcctccctgttcatcattgcctccaaggtGtgagtgccgaaaactgcccggcccctggccctgtgtgtgttggggtggggagggacagctgtcttgccatccccctggccattggtcccttttggtgggttccgagtgaaccatggtttgtggatcaggtcagtacagctgccctagactgtttccatgcctagcagcaaataaggttgactgatgaaatcctgaacacatcagtttgaacctttgtgtcctgaccacacccgctcgccttgcggcatctctgctcagcctgggactgctcgccactttcttacaaacctgtcctttatgcctgctccttttcttgttgttgtttttgagacagagtctggctctgtcacccaggctggagtgtagtggcgtgatctcggctcactgcaatctccgtctcccaggtttcaagcgattctcctgcctcagcctcccaagtagctgggactaggcacacaccaccacgcctggctaattttattatttatttatttattattattattttttagtagagatgggcttttgccatgttggccatgctggtcttgaactcctgacctcaggtgatgcacccccccttggcctcccaaagtgctgggattacagtcttgagccgccatgcccagccatgcttgctcctttctgatcccagagccatgccctgataatagaggggttttgtttgtttttatttatgttctatttactcacatgaatgtacataaaatatgaagaattccatctgaattctgagataccttccagcctgggccgaagagagcctcaggttgactgcagcccctcaggggagaagctgcggcctttgacctgcaggcttagggttggggggtgtgtttaccgtcccccctcctctggttttaaagagctggaatctcaggaggttatgtggcgtcactgtcactgacctgcaaatactgctccatgggacagctgggcattgccttggcctctactgctgaaccctggctcaaggcctgcacccacccctaagctcgggcgcccactgctgttctctttggttgcagacctttactacccaggagaccatcacgaatgcagagacggcgaaggagtggtttctccaggcggccaaggat

**4. Normal transcriptional result sequence as expected**

PCR amplification of normal group expected sequence of 374bp: (upper-case text = vector transcript sequence; lower-case text = target gene exon transcript sequence)

GGCTAACTAGAGAACCCACTGCTTACTGGCTGCTAGCGTTTAAACTTAAGCTTGGTACCGAGCTCGGATCC cgtgtccggagcggtgactggaaggggtacacaggcaagaccatcacggacgtcatcaacattggcattggcggctccgacctgggacccctcatggtgactgaagcccttaagccatactcttcaggaggtccccgcgtctggtatgtctccaacattgatggaactcacattgccaaaaccctggcccagctgaaccccgagtcctccctgttcatcattgcctccaagacctttactacccaggagaccatcacgaatgcagagacggcgaaggagtggtttctccaggcggccaaggat

**5. Sanger sequencing of the RT-PCR products of WT:**

CGTGTCCGGAGCGGTGACTGGAAGGGGTACACAGGCAAGACCATCACGGACGTCATCAACATTGGCATTGGCGGCTCCGACCTGGGACCCCTCATGGTGACTGAAGCCCTTAAGCCATACTCTTCAGGAGGTCCCCGCGTCTGGTATGTCTCCAACATTGATGGAACTCACATTGCCAAAACCCTGGCCCAGCTGAACCCCGAGTCCTCCCTGTTCATCATTGCCTCCAAGACCTTTACTACCCAGGAGACCATCACGAATGCAGAGACGGCGAAGGAGTGGTTTCTCCAGGCGGCCAAGGAT

**6. Sanger sequencing of the RT-PCR products of  c.633+3A>G variant：r. 546_633del:**

CGTGTCCGGAGCGGTGACTGGAAGGGGTACACAGGCAAGACCATCACGGACGTCATCAACATTGGCATTGGCGGCTCCGACCTGGGACCCCTCATGGTGACTGAAGCCCTTAAGCCATACTCTTCAGGAGGTCCCCGCGTCTGACCTTTACTACCCAGGAGACCATCACGAATGCAGAGACGGCGAAGGAGTGGTTTCTCCAGGCGGCCAAGGAT

**7. Sanger sequencing of the RT-PCR products of c.633+3A>G variant：r. 633+1_633+2insGT:**

CGTGTCCGGAGCGGTGACTGGAAGGGGTACACAGGCAAGACCATCACGGACGTCATCAACATTGGCATTGGCGGCTCCGACCTGGGACCCCTCATGGTGACTGAAGCCCTTAAGCCATACTCTTCAGGAGGTCCCCGCGTCTGGTATGTCTCCAACATTGATGGAACTCACATTGCCAAAACCCTGGCCCAGCTGAACCCCGAGTCCTCCCTGTTCATCATTGCCTCCAAGGTACCTTTACTACCCAGGAGACCATCACGAATGCAGAGACGGCGAAGGAGTGGTTTCTCCAGGCGGCCAAGGAT

**8. Sanger sequencing of the RT-PCR products of c.633+3A>G variant：WT**

CGTGTCCGGAGCGGTGACTGGAAGGGGTACACAGGCAAGACCATCACGGACGTCATCAACATTGGCATTGGCGGCTCCGACCTGGGACCCCTCATGGTGACTGAAGCCCTTAAGCCATACTCTTCAGGAGGTCCCCGCGTCTGGTATGTCTCCAACATTGATGGAACTCACATTGCCAAAACCCTGGCCCAGCTGAACCCCGAGTCCTCCCTGTTCATCATTGCCTCCAAGACCTTTACTACCCAGGAGACCATCACGAATGCAGAGACGGCGAAGGAGTGGTTTCTCCAGGCGGCCAAGGAT

**9. PCR conditions**

The PCR amplification conditions were as follows: a denaturation step at 94°C for 2 min, followed by 35 cycles of denaturing at 98°C for 10s, annealing for 30s at 62°C, extension at 68°C for 1.5 min.

**10. Supplementary Table S1. Primer sequences used to amplify GPI genomic fragments**

**Supplementary Table S1. Primer sequences used to amplify *GPI* genomic fragments**

| **Process** | **Name of Prime** | **Primer sequence (5’→3’)** |
| --- | --- | --- |
| Amplify the pMini- *GPI*- WT | *GPI*-F | AAGCTTGGTACCGAGCTCGGATCCCGTGTCCGGAGCGGTGACTGGAAGGGG |
|  | *GPI*-R | TTAAACGGGCCCTCTAGACTCGAGATCCTTGGCCGCCTGGAGAAACCACTCC |
| Amplify the pMini- *GPI*- c.633+3A>G mutant | *GPI*-c.633+3A>G-F | AAGGTgTGAGTGCCGAAAACTGCCCGGCCCCT |
|  | *GPI-*c.633+3A>G-R | TTCGGCACTCAcACCTTGGAGGCAATGATGAACA |
