# Supplementary material for: The novel compound heterozygous variants identified in a Chinese family with glucose phosphate isomerase deficiency and pathogenicity analysis
Source: BMC Med Genomics. 2023 Jul 10;16:162. doi: 10.1186/s12920-023-01603-x (PMC10332073; doi:10.1186/s12920-023-01603-x)
Supplement: Supplementary file 2 — Supplementary Material 2 [file 12920_2023_1603_MOESM2_ESM.docx]

**Supplementary material 2.**

**Wild-type protein sequence：**

MAALTRDPQFQKLQQWYREHRSELNLRRLFDANKDRFNHFSLTLNTNHGHILVDYSKNLVTEDVMRMLVDLAKSRGVEAARERMFNGEKINYTEGRAVLHVALRNRSNTPILVDGKDVMPEVNKVLDKMKSFCQRVRSGDWKGYTGKTITDVINIGIGGSDLGPLMVTEALKPYSSGGPRVWYVSNIDGTHIAKTLAQLNPESSLFIIASKTFTTQETITNAETAKEWFLQAAKDPSAVAKHFVALSTNTTKVKEFGIDPQNMFEFWDWVGGRYSLWSAIGLSIALHVGFDNFEQLLSGAHWMDQHFRTTPLEKNAPVLLALLGIWYINCFGCETHAMLPYDQYLHRFAAYFQQGDMESNGKYITKSGTRVDHQTGPIVWGEPGTNGQHAFYQLIHQGTKMIPCDFLIPVQTQHPIRKGLHHKILLANFLAQTEALMRGKSTEEARKELQAAGKSPEDLERLLPHKVFEGNRPTNSIVFTKLTPFMLGALVAMYEHKIFVQGIIWDINSFDQWGVELGKQLAKKIEPELDGSAQVTSHDASTNGLINFIKQQREARVQ*

**Protein sequence of Variant(NM_000175.5：c.546_633del):p.Trp182X**

MAALTRDPQFQKLQQWYREHRSELNLRRLFDANKDRFNHFSLTLNTNHGHILVDYSKNLVTEDVMRMLVDLAKSRGVEAARERMFNGEKINYTEGRAVLHVALRNRSNTPILVDGKDVMPEVNKVLDKMKSFCQRVRSGDWKGYTGKTITDVINIGIGGSDLGPLMVTEALKPYSSGGPRV*

**Protein sequence of Variant(NM_000175.5：c.633+1_633+2insGT)：p.Thr212ValfsTer43**

MAALTRDPQFQKLQQWYREHRSELNLRRLFDANKDRFNHFSLTLNTNHGHILVDYSKNLVTEDVMRMLVDLAKSRGVEAARERMFNGEKINYTEGRAVLHVALRNRSNTPILVDGKDVMPEVNKVLDKMKSFCQRVRSGDWKGYTGKTITDVINIGIGGSDLGPLMVTEALKPYSSGGPRVWYVSNIDGTHIAKTLAQLNPESSLFIIASKVPLLPRRPSRMQRRRRSGFSRRPRILLQWRSTLLPCLLTQPK*
