# Supplementary material for: The novel compound heterozygous variants identified in a Chinese family with glucose phosphate isomerase deficiency and pathogenicity analysis
Source: BMC Med Genomics. 2023 Jul 10;16:162. doi: 10.1186/s12920-023-01603-x (PMC10332073; doi:10.1186/s12920-023-01603-x)
Supplement: Supplementary file 3 — Supplementary Material 3 [file 12920_2023_1603_MOESM3_ESM.docx]

**Supplementary material 3.**

**Title: Original graphs of PCR product on agarose gels**

**Legend: original graphs of PCR product of NM_000175.5：c.633+3A>G and WT in *GPI***

The original images are presented below:


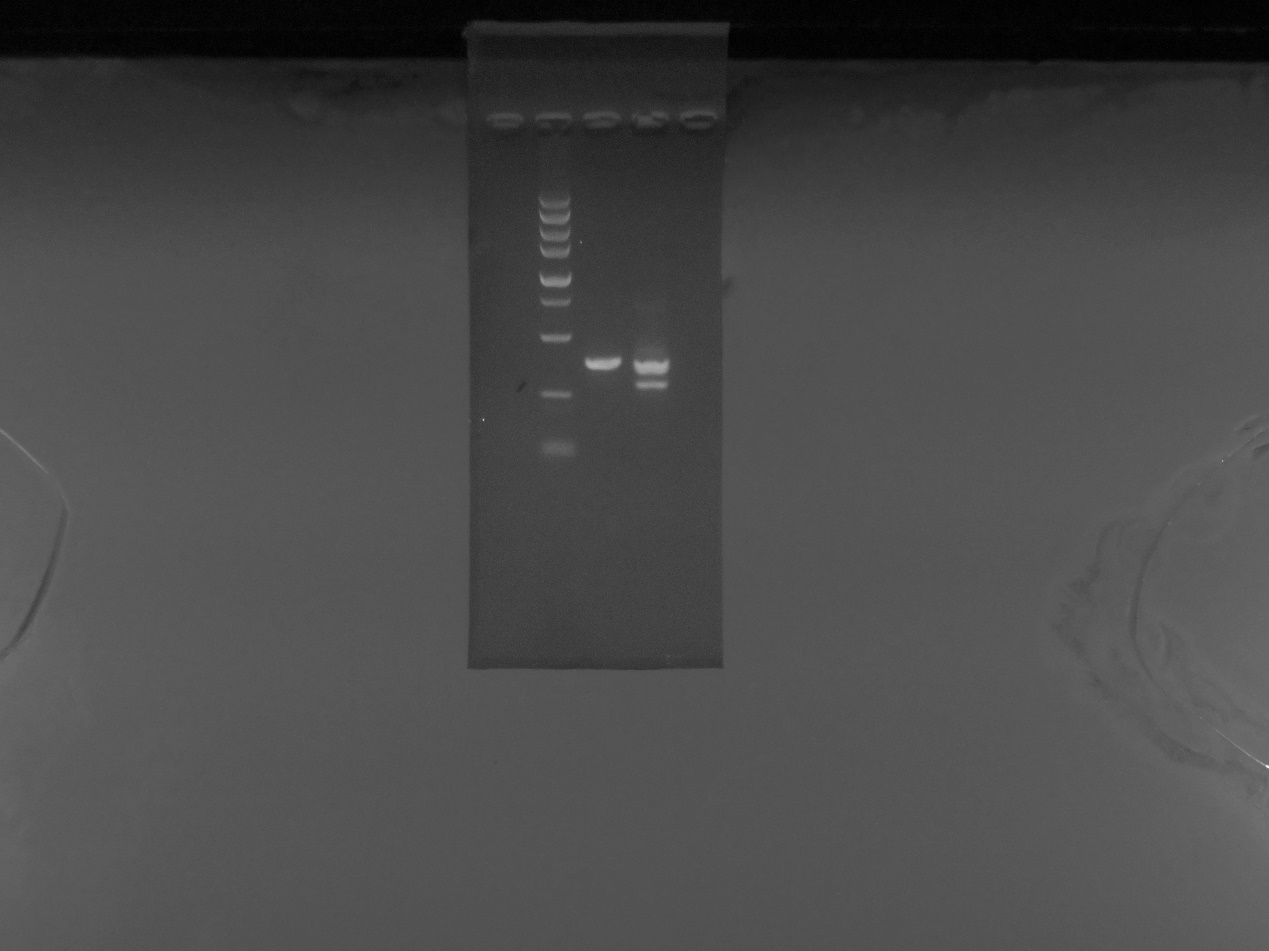


**Supplementary Figure 1.** **Original graphs of PCR product on agarose gels**

A)original graphs of PCR product of NM_000175.5：c.633+3A>G and WT in *GPI* gene
